# Cardiac dysfunction after acute ischaemic stroke: Long-term outcomes from the SICFAIL cohort

**DOI:** 10.1016/j.ijcha.2026.101926

**Published:** 2026-04-20

**Authors:** Kathrin Ungethüm, Felipe A. Montellano, Viktoria Rücker, Timo Ludwig, Charles D.A. Wolfe, Caroline Morbach, Stefan Frantz, Stefan Störk, Christoph Kleinschnitz, Karl Georg Haeusler, Peter U. Heuschmann, for the SICFAIL study group

**Affiliations:** a Institute of Clinical Epidemiology and Biometry, University of Würzburg, Würzburg, Germany; b Institute for Medical Data Sciences, University Hospital Würzburg, Würzburg, Germany; c Department of Neurology, University Hospital Würzburg, Würzburg, Germany; d Department of Population Health Sciences, King’s College London, London, United Kingdom; e Department of Epidemiology and Clinical Research, Comprehensive Heart Failure Center, University of Würzburg, Würzburg, Germany; f Department of Internal Medicine I, University Hospital Würzburg, Würzburg, Germany; g Department of Neurology, University Hospital Essen, Essen, Germany; h Department of Neurology, University Hospital Ulm, Ulm, Germany

**Keywords:** Ischaemic Stroke, Stroke Outcome, Systolic Dysfunction, Heart Failure

## Abstract

**Background:**

Systolic dysfunction, diastolic dysfunction, and clinically overt heart failure are frequently encountered after acute ischaemic stroke. We investigated whether these cardiac phenotypes, considered as distinct entities, are associated with readmission and death within two years after stroke in the prospective SICFAIL cohort.

**Methods:**

Adults with acute ischaemic stroke were consecutively enrolled between 01/2014 and 02/2017. Cardiac function was assessed at baseline, and patients were followed annually by mail or telephone. The primary endpoint was the composite of all-cause readmission or death. Secondary analyses considered individual endpoints and cardiovascular readmissions. Associations were estimated using multivariable Cox proportional hazards models.

**Results:**

Of 696 enrolled patients, 644 (92.5%) had interpretable echocardiographic data. During two-year follow-up, 206 of 554 patients (37.1%) with complete outcome information were rehospitalised, and 63 of 577 patients (11.4%) with available vital status data died. After adjustment, systolic dysfunction and clinically overt heart failure were independently associated with the composite endpoint (systolic dysfunction: hazard ratio [HR] 1.97 (95% confidence interval [CI], 1.34–2.91); clinically overt heart failure: HR 1.62, 95% CI 1.02–2.58). Systolic dysfunction also predicted cardiovascular readmissions (HR 2.27, 95% CI 1.22–4.21). Diastolic dysfunction was not associated with adverse outcomes.

**Conclusion:**

In this cohort, systolic dysfunction and clinically overt heart failure at the time of ischaemic stroke independently predicted the composite of readmission or death over the subsequent two years, whereas isolated diastolic dysfunction was not prognostically informative. Routine echocardiographic assessment after stroke may therefore help identify patients who would benefit from intensified cardiac follow‑up and secondary prevention.

## 1 Introduction

Increasing evidence suggests a bidirectional relationship between the brain and heart, facilitated by the autonomic nervous system, inflammatory processes, and humoral stress responses[1], [2], [3]. This association is particularly pronounced following injury to either or both organs. In survivors of ischaemic stroke (IS), cardiac abnormalities are common: studies suggest that roughly one in ten patients develops systolic dysfunction (SD), around a quarter show signs of diastolic dysfunction (DD), and up to five percent meet diagnostic criteria for clinically overt heart failure (coHF)[4]. These derangements correlate with poorer functional recovery, greater disability and a higher incidence of adverse cardiovascular events and death in the months immediately following a stroke[5]. While the course after stroke in patients with chronic heart failure has been investigated in short- and long-term perspective[5], [6], most existing analyses regarding distinct contributions of SD, DD in contrast to coHF defined at the time of the index stroke, however, have concentrated on short‑term outcomes[7], [8], [9], [10] and long-term prognostic data are sparse.

To address this gap, we studied participants in the Stroke–Induced Cardiac Failure (SICFAIL) cohort—an investigator–initiated, prospective, single–centre study. We sought to determine whether SD, DD or coHF present in IS patients independently predict all‑cause readmission and all‑cause mortality over a two‑year period. Secondary objectives were to explore associations between these cardiac phenotypes and recurrent stroke, cardiovascular-specific readmission and mortality.

## 2 Methods

The SICFAIL study is an investigator-initiated hospital-based single-center prospective cohort study, aiming to investigate prevalence and course of cardiac function after IS (clinical trial registration: DRKS00011615). The study design was described in detail elsewhere[4]. In brief, patients with acute IS were recruited at a supra-regional Stroke Unit, at Department of Neurology, University Hospital Würzburg, Germany between 01/2014 and 02/2017. All patients or their legal representatives provided written informed consent before study inclusion. The study was approved by the Ethics Committee of the Medical Faculty at University of Würzburg (vote 176/13, 22.08.2013) and complies with the Declaration of Helsinki. Detailed in- and exclusion criteria and data collection processes are given in the *eMethods*. Baseline assessments comprised standardized transthoracic echocardiography[11], performed at a median of 4 days after admission (IQR 2–5)[4]; measurement of blood-based biomarkers using fasting blood samples obtained on the morning after study enrolment (median day 3, IQR (2–4)[4]; a structured interview on medical history; and review of the medical records. Diagnostic criteria for SD, DD and coHF are given in Table 1. As more than 95% of DD patients within the SICFAIL cohort had Grade 2 or Grade 3 DD[4], no distinction of DD grades was made due to power issues. First and second year follow-up were conducted by phone or mail, and standardi**s**ed questionnaires were used. If a patient could not be reached, information on vital status was collected through a patient’s next of kin or the local resident’s registration office.

**Table 1:** Definition of systolic dysfunction, diastolic dysfunction and clinically overt heart failure in SICFAIL (modified from Heuschmann et al.[4]).

**Type ofcardiacdysfunction**	**Echocardiography & Biomarker**		**Signs & Symptoms**
Systolic dysfunction[29]	Left ventricular ejection fraction < 52% in men < 54% in women		−
		
Diastolic dysfunction[31]	Left ventricular ejection fraction ≥ 52% in men ≥ 54% in women AND ≥ 3 of the following criteria: • LAVI > 34 mL/m2 OR LA area > 30 cm2 (if LAVI not available) • Average E/e’ > 14 • Lateral e’ < 10 cm/s OR septal e’ < 7 cm/s • Tricuspid regurgitation maximal flow velocity > 2.8 m/s		−
		
Clinically overt heart failure[30]	Combination of echocardiography, laboratory findings and clinically relevant signs and symptoms		
	*Heart failure with preserved ejection fraction (HFpEF)* Left ventricular ejection fraction ≥ 50% AND NT-proBNP ≥ 125 mg/dl	**A** **N** **D**	At least 2 major criteria or 1 major and 2 minor criteria: *Major criteria* • Pre-stroke paroxysmal nocturnal dyspnoea • Pre-stroke orthopnoea (sleeping with the upper body at an angle > 45°) • Pre-stroke dyspnoea on mild exertion • Rales on admission • Acute pulmonary oedema (chest X-ray) • Cardiomegaly (LVED > 58.4 mm (men) or > 52.2 mm (women)) • Third heart sound on admission *Minor criteria* • Pre-stroke lower limb oedema or as assessed on admission • Pleural effusion (chest X-ray) • Heart rate ≥ 120b.p.m. at day 3 post-stroke • Nycturia (>2 times/night) • Pre-stroke dyspnea on moderate or intense exertion
	*Heart failure with preserved ejection fraction (HFmrEF)* Left ventricular ejection fraction 40–49% AND NT-proBNP ≥ 125 mg/dl		
	*Heart failure with reduced ejection fraction (HFrEF)* Left ventricular ejection fraction < 40%		

The study’s primary endpoint was a composite of all-cause rehospitalisation and all-cause mortality within two years after stroke. Secondary endpoints were all-cause mortality, and readmission, subdivided into all-cause, CVD, stroke and heart diseases.

### 2.1 Statistical Methods

We performed a complete case analysis. Baseline characteristics are presented in n (%) or median (Interquartile Range (IQR)) and compared using χ^2^-test and Wilcoxon rank-sum tests, as appropriate. The association of different exposures with the combined endpoint was investigated using Cox proportional hazard models with Breslow ties and reported as Hazard Ratios (HR) with 95% confidence intervals (95% CI). The basic model included clinically established variables with impact on outcomes after stroke (age at admission, stroke severity (National Institute of Health Stroke Scale (NIHSS) score[12]) and pre-stroke dependency[13], [14]) (Model 1). The fully adjusted model additionally included stroke aetiology (suspected cardioembolic vs. non-cardioembolic), and clinically relevant comorbidities (history of coronary heart disease, previous stroke or transient ischemic attack (TIA), hypertension, diabetes, dyslipidaemia, atrial fibrillation) (Model 2). SD, DD, and coHF were separately added to the models. Secondary endpoints were analysed using Model 1 (all-cause mortality, disease-specific readmissions) or Model 2 (all-cause readmission). Time under risk was respected in months. Consequently, time to event was set to one month, in case, a patient was readmitted or died within the same month as the index stroke.

Sensitivity analyses investigated the impact of high-sensitivity Troponin (hs-TnT) in Model 2. Moreover, potential effect modifications by sex, coronary heart disease, and cardiovascular risk factors (hypertension, dyslipidaemia, and diabetes) were analysed applying Model 1.

Statistical significance was set at α = 0.05 (two-tailed). Statistical analysis was performed using SAS 9.4 (SAS Institute Inc., Cary, NC, USA) and R 4.3.1 (RFoundation for Statistical Computing, Vienna, Austria).

## 3 Results

### 3.1 Participants & occurrence of the primary outcome

**Fig. 1:**
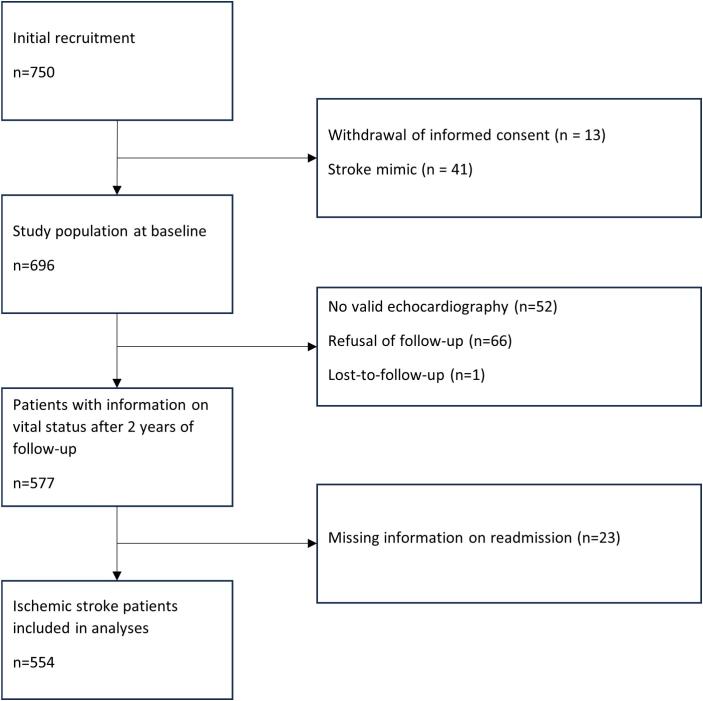
Participant’s flow diagram.

Initially, 750 patients with symptoms suggestive of IS were included in the SICFAIL study. The baseline study population consisted of 644 patients with confirmed diagnosis of IS and interpretable echocardiographic data. Information on the combined endpoint after one and/or two years could be obtained in 554 (86.0%) patients (Fig. 1). Median age was 70.5 years (IQR 60.0–78.0), 61.9% male, and median NIHSS score on admission 3 IQR (1–5) points. In the study population considered for analyses on hand, SD was prevalent in 8.1%, DD in 22.7%, and coHF in 5.0%, respectively. Of patients with SD, 20% fulfilled the diagnostic criteria of coHF, as did 11.9% of patients with DD. Vital status after two years of follow-up was available for 577 patients (89.6%). The combined endpoint (all-cause mortality or all-cause readmission) occurred in 269 of 554 (48.6%) patients. Within two years of follow-up, 63 (11.4%) patients died, and 206 (37.2%) patients were readmitted at least once. Of those readmitted, seven patients were readmitted due to heart failure, 27 suffered other cardiac events excluding any type of stroke, 55 patients were diagnosed with recurrent IS or TIA, one patient was readmitted due to intracerebral haemorrhage, and 135 patients were readmitted for other diagnoses (Supplemental Table S1). For eight patients, who died during follow-up, previous hospitalisation was reported.

### 3.2 Baseline characteristics

Baseline characteristics of the study population are given in Table 2. In comparison to patients without outcome occurrence, patients developing a combined endpoint during follow-up were older, more likely to be depended pre-stroke, and had a slightly higher median stroke severity. Moreover, patients with and without endpoint occurrence differed in the prevalence of comorbidities and laboratory measures. SD and coHF were more prevalent in patients who developed the primary endpoint. A comparison of patients with and without different forms of cardiac dysfunction in the SICFAIL study was published previously[4].

**Table 2:** Baseline characteristics of the study population.

	**All** **(n = 554)**	**Readmission / Mortality** **(n = 269)**	**Noreadmission** **(n = 285)**
Age, median (IQR)	70.5 (60.0–78.0)	72.0 (62.0–80.0)	68.0 (58.0–78.0)
<55 years, n (%)	88 (15.9)	30 (11.2)	58 (20.4)
55–64 years, n (%)	100 (18.1)	50 (18.6)	50 (17.5)
65–74 years, n (%)	145 (26.2)	69 (25.7)	76 (26.7)
75–84 years, n (%)	166 (30.0)	84 (31.2)	82 (28.8)
≥85 years, n (%)	55 (9.9)	36 (13.4)	19 (6.7)
Male, n (%)	343 (61.9)	170 (63.2)	173 (60.7)
***Lifestyle***			
Dependency, pre-stroke, n (%)	65 (12.0)	41 (15.5)	24 (8.7)
% missing	2.4		
Regular physical activity, n (%)	273 (50.1)	112 (42.1)	161 (57.7)
% missing	1.6		
Ever smoking, n (%)	295 (54.1)	141 (53.2)	154 (55.0)
% missing	1.6		
Smoking at baseline, n (%)	106 (19.5)	49 (18.5)	57 (20.4)
% missing	1.6		
***Indexevent***			
NIHSS score, median (IQR)	3 (1–5)	3 (2–5)	3 (1–5)
≤4 points	402 (72.6)	189 (70.3)	213 (74.7)
5–15 points	135 (24.4)	69 (25.7)	66 (23.2)
≥16 points	17 (3.1)	11 (4.1)	6 (2.1)
Thrombolysis, n (%)	109 (19.7)	48 (17.8)	61 (21.4)
Mechanical thrombectomy, n (%)	7 (1.3)	2 (0.8)	5 (1.8)
% missing	0.2		
TOAST classification, n (%)			
Large-artery atherosclerosis,	64 (11.6)	35 (13.0)	29 (10.2)
Cardioembolic stroke	161 (29.1)	91 (33.8)	70 (24.6)
Small vessel occlusion	81 (14.6)	30 (11.2)	51 (17.9)
Stroke of other determined cause	44 (7.9)	19 (7.1)	25 (8.8)
Undetermined aetiology	203 (36.6)	94 (34.9)	109 (38.3)
% missing	0.2		
Time from onset to admission > 6 h, n (%)	259 (46.8)	135 (50.2)	124 (43.5)
Insular lesion, n (%)	72 (13.0)	39 (14.5)	33 (11.6)
Glucose at admission, mg/dl, median (IQR)	112 (97–135)	114 (98–144)	111 (97–128)
% missing	2.2		
High-sensitive Troponin T ≥ 14 ng/l, n (%)	164 (36.9)	108 (50.0)	56 (24.6)
% missing	19.9		
NT-proBNP ≥ 125 pg/ml, n (%)	296 (66.5)	161 (74.5)	135 (59.0)
% missing	19.7		
***Comorbidities / Medicalhistory***			
Atrial fibrillation, n (%)	119 (21.5)	74 (27.5)	45 (15.8)
Hypertension, n (%)	418 (75.9)	220 (82.1)	198 (70.0)
No medication	42 (7.6)	22 (8.2)	20 (7.1)
With medication	376 (68.2)	198 (73.9)	178 (62.9)
% missing	0.5		
Hypercholesterinaemia or LDL > 130 mg/dl at baseline, n (%)	360 (68.8)	182 (72.8)	178 (65.2)
No medication	170 (32.5)	76 (30.4)	94 (34.4)
With medication	190 (36.6)	106 (42.4)	84 (30.8)
% missing	5.6		
Diabetes, n (%)	152 (28.3)	93 (35.8)	59 (21.3)
No medication, n (%)	52 (9.7)	34 (13.1)	18 (6.5)
With medication, n (%)	101 (18.8)	59 (22.7)	42 (15.1)
% missing	2.9		
History in stroke / TIA, n (%)	128 (23.4)	77 (29.1)	51 (18.2)
% missing	1.4		
Coronary heart disease, n (%)	91 (16.7)	57 (21.7)	34 (12.1)
% missing	1.8		
***Cardiac dysfunction***			
Systolic dysfunction, n (%)	45 (8.1)	33 (12.3)	12 (4.2)
LVEF < 30%	3 (6.7)	3 (9.1)	0 (0.0)
Diastolic dysfunction*, n (%)	109 (22.7)	53 (24.1)	56 (21.5)
% missing	5.7		
Clinically overt heart failure, n (%)	33 (6.0)	24 (8.9)	9 (3.2)
HFpEF	26 (4.7)	17 (6.3)	9 (3.2)
HFmrEF	2 (0.4)	2 (0.7)	0 (0)
HFrEF	5 (0.9)	5 (1.9)	0 (0)

*in patients without systolic dysfunction; HFpEF: Heart failure with preserved ejection fraction; HFmrEF: Heart failure with mid-range ejection fraction; HFrEF: Heart failure with reduced ejection fraction

### 3.3 Non-responder analysis

Patients who were lost to follow-up or had incomplete outcome data (n = 90) did not differ from patients with follow-up information regarding demographic characteristics, pre-stroke dependency, stroke severity, and stroke aetiology but had statistically significant higher proportions of prolonged admission times, history of diabetes and SD (Supplemental Table 2).

### 3.4 Primary endpoint

Univariate associations are given in Supplemental Table 3. Patients with SD or coHF at baseline had consistently higher risks of all-cause readmission or all-cause mortality after adjustment for baseline characteristics and comorbidities with HR = 1.97 (95% CI, 1.34–2.91) for SD and HR = 1.62 (95% CI, 1.02–2.58) for coHF (Table 3A*,* Supplemental Fig. 1). DD was not associated with the combined endpoint after adjustment (HR = 0.88 (95% CI, 0.61–1.27)). In the fully adjusted model, history of stroke or TIA was associated with the combined endpoint independently of cardiac function (HR = 1.41 (95% CI, 1.06–1.87)). History of coronary heart disease was also independently associated with the combined endpoint (HR = 1.45 (95% CI, 1.06–1.99)). After adding SD to the model, history in coronary heart disease was not associated with the combined endpoint anymore (Supplemental Table 4).

**Table 3:** Association of different types of cardiac dysfunction with all-cause mortality and all-cause readmission (HR, 95% CI).

**A. Analyses of the primary endpoint (all-cause readmission or all-cause mortality)**		
	**Model 1**^a^	**Model 2**^b^
Systolic dysfunction	**2.07 (1.43**–**3.01)**	**1.97 (1.34**–**2.91)**
Diastolic dysfunction	0.96 (0.68–1.37)	0.88 (0.61–1.27)
Clinically overt heart failure	**1.90 (1.23**–**2.92)**	**1.62 (1.02**–**2.58)**
		
**B. Analyses of secondary endpoints**		
	**Model 1**^a^	**Model2**^b^

**All-causemortality**		
Systolic dysfunction	1.34 (0.78–3.44)	−
Diastolic dysfunction	1.10 (0.57–2.12)	−
Clinically overt heart failure	1.47 (0.63–3.45)	−
**All-causereadmission**		
Systolic dysfunction	**1.95 (1.27**–**2.98)**	**1.94 (1.24**–**3.05)**
Diastolic dysfunction	0.93 (0.63–1.37)	0.94 (0.62–1.42)
Clinically overt heart railure	**1.79 (1.10**–**2.93)**	**1.74 (1.03**–**2.96)**
**Readmission for CVD**		
Systolic dysfunction	**2.27 (1.22**–**4.21)**	−
Diastolic dysfunction	1.45 (0.78–2.72)	−
Clinically overt heart failure	1.25 (0.54–2.92)	−
**Readmission for acardiacevent**		
Systolic dysfunction	2.18 (0.75–6.32)	−
Diastolic dysfunction	1.25 (0.42–3.70)	−
Clinically overt heart failure	2.32 (0.68–7.89)	−
**Stroke-relatedreadmission**		
Systolic dysfunction	1.74 (0.78–3.86)	−
Diastolic dysfunction	1.61 (0.78–3.31)	−
Clinically overt heart failure	1.06 (0.38–2.99)	−

a adjusted for age (per year), NIHSS at admission (per point), dependency pre-stroke (yes vs. no).

b adjusted for Model 1, history in stroke/TIA, history in coronary heart disease (myocardial infarction/angina pectoris), atrial fibrillation, hypertension, dyslipidaemia, diabetes, cardioembolic stroke aetiology.

### 3.5 Secondary endpoints

The analyses of secondary endpoints are presented in Table 3B. Whereas an association for DD was observed in univariate analysis, none of the different types of cardiac dysfunction was associated with all-cause mortality after adjustment. Readmission was associated with SD (HR = 1.94 (95% CI, 1.24–3.05)) and coHF (HR = 1.74 (95% CI, 1.03–2.96)) after adjustment for baseline characteristics, stroke aetiology and comorbidities, but not with DD (HR = 0.94 (95% CI, 0.62–1.42)). Moreover, patients with SD were at increased risk for readmission due to CVD after adjustment for baseline characteristics (HR = 2.27 (95% CI, 1.22–4.21)). After differentiation of readmission due to a cardiac event or ischaemic/haemorrhagic stroke, no statistically significant association was observed for SD (data not shown).

### 3.6 Sensitivity analyses

Elevated levels of hs-TnT (≥14 ng/l) attenuated the association of both, SD (HR = 1.69 (95% CI, 1.11–2.58)), and coHF with the combined endpoint (HR = 1.53 (95% CI, 0.95–2.46)). A statistically significant interaction of sex and SD was observed (p = 0.03), suggesting an increased risk for the combined endpoint in men with SD (HR = 2.81 (95% CI, 1.84–4.30)) but not in women (HR = 0.98 (95% CI, 0.43–2.23)). No interactions of SD with history in coronary heart disease or cardiovascular risk factors were observed. Exclusion of patients with imputed date of the outcome occurrence (n = 31) due to missing information had no substantial impact on the effect estimates of SD (HR = 1.98 (95% CI, 1.32–2.98)), DD (HR = 0.93 (95% CI, 0.63–1.37)), and coHF (HR = 1.69 (95% CI, 1.05–2.70)) after adjustment.

## 4 Discussion

In this prospective cohort of predominantly mild IS survivors, we observed that SD and coHF present in post-acute stroke nearly doubled the risk for all-cause mortality or hospital readmission over two years. This association persisted after adjustment for age, stroke severity, pre-stroke dependency and major cardiovascular comorbidities. Secondary analyses revealed that both SD and coHF primarily drove the risk of readmission rather than death, and that SD was specifically associated with a two‑fold increase in cardiovascular readmissions. By contrast, DD showed no independent association with any outcome. Recurrent stroke at baseline and a history of coronary heart disease remained important covariates, underscoring the complex interplay of cerebrovascular and cardiac pathology. Together, these observations suggest that ventricular pump failure, whether asymptomatic or symptomatic[4], at the time of the index stroke represents a powerful long-term prognostic marker.

Previous studies report an increased risk of mortality in patients with SD in the general population[15], [16] and describe SD as risk factor for IS[17]. Moreover, recent studies in IS patients report an increased risk for unfavourable functional outcome and mortality after three months and one year in patients with SD[7], [8], [18]. The analyses on hand as well as previously published data suggest an association of SD and coronary heart disease[4], [19]. At baseline, 35.6% of patients with SD reported a prior history of myocardial infarction or angina pectoris[4], both of which might have contributed to an increased risk of recurrent events and mortality[20], [21]. Nevertheless, owing to the study design, pre-stroke cardiac function was largely unknown; therefore, the present data do not permit conclusions as to whether IS itself induced alterations in cardiac function. Notably, 22.6% of patients with SD, 10.9% of patients with DD, and 37.1% of patients with coHF reported a history of heart failure, although the underlying diagnostic criteria were unavailable[4].

Asymptomatic patients with SD might be at increased risk to develop symptomatic heart failure subsequently[15], however, data on progression from SD to coHF with impaired ejection fraction in patients with history in IS is scarce and several comorbidities and conditions might contribute to this progression[22]. Although, within the SICFAIL study no clinical cardiac follow-up examination was performed to confirm this hypothesis. Readmission rates due to heart failure were low in the investigated population, but the development of coHF is a long-term process, and, moreover, heart failure symptoms do not cause immediate hospital admission in many patients[23].

In the SICFAIL study population, DD was not associated with all-cause mortality, all-cause or CVD-related readmission. A previous study in IS patients with predominantly mild stroke found a significantly increased risk of major cardiovascular events and recurrent IS in patients with DD, but no statistically significant association with all-cause mortality[9]. Although the study population was similar to the SICFAIL study population in terms of stroke severity, the study only included patients with suspected non-cardioembolic stroke and SD was not regarded as a separate entity of cardiac dysfunction. Patients with left ventricular DD had a LVEF mean value of 52.4%. In contrast, patients with SD (LVEF < 52% (men), <54% (women)) were excluded from the definition of DD in the SICFAIL study. The differences between the underlying study populations might have contributed to different results.

Heart failure is one of the main risk factors for unfavourable short- and long-term outcome after IS in terms of functional outcome, mortality, and recurrent major CV events[5], [6], [10], [24], [25], [26]. In the SICFAIL study population, coHF was associated with the combined endpoint and all-cause readmission within two years after IS. Probably due to low event numbers, more specific secondary endpoint analyses, including cardiovascular readmissions, revealed statistically non-significant, and underpowered analyses.

Inclusion of hs-TnT to the analysis attenuated the association of SD and coHF with the combined endpoint. This is in line with previous findings from the SICFAIL study that demonstrated that SD is associated with increased hs-TnT levels in IS patients[27]. The observation that troponin levels attenuated the impact of SD and coHF on outcomes raises the possibility that biomarker‑guided therapy could refine risk stratification. A third sensitivity analysis focused on a potential interaction of cardiac dysfunction and sex, as increasing evidence suggests that heart-brain interactions might differ substantially in women and men[28]. The analyses of the SICFAIL study suggest a stronger impact of SD on the primary outcome in men, however, the results must be interpreted very carefully due to very low case numbers in women. Larger trials are needed to investigate this association, and potential biological and social determinants in more detail and precision.

Our findings have implications for practice. First, they underscore the importance of comprehensive cardiac assessment in the acute phase of stroke, including echocardiography and biomarkers. Identification of SD or coHF should prompt early involvement of cardiologists and the initiation or optimisation of adequate heart failure therapy. Second, stroke survivors with these abnormalities merit closer monitoring after discharge, with a low threshold for referral to heart failure services. Given the strong association with readmissions—many of which were cardiovascular—structured follow‑up and rehabilitation programmes could reduce hospitalisations and improve quality of life. Third, the lack of association between DD and outcomes suggests that not all echocardiographic abnormalities confer the same risk; clinicians should therefore differentiate between systolic and diastolic impairment.

## 5 Limitations

The SICFAIL study prospectively recruited consecutive stroke patients and obtained detailed echocardiographic phenotyping at baseline, ensuring accurate classification of cardiac function. Follow‑up rates were high, vital status was confirmed through residents’ registry offices, and all analyses adjusted for key confounders. The cohort’s mild stroke severity, while limiting generalizability, allowed us to isolate the impact of cardiac dysfunction without the confounding effects of severe neurological disability.

Nonetheless, limitations must be acknowledged. As a single‑centre study conducted in an urban–rural region, the findings may not apply to other healthcare systems or populations with different stroke subtypes and comorbidity patterns. Selection bias is possible, as patients with severe strokes or impaired consciousness were less likely to provide informed consent. Information on readmissions preceding death was incomplete, leading to potentially underestimating the association of cardiac dysfunction with the composite endpoint. Our definition of DD excluded patients with mildly reduced ejection fraction to distinguish it from SD, which may limit comparability with other studies. To comply with the baseline findings of the SICFAIL study, we applied the 2016 European guidelines for diagnosis of coHF instead of the universal definition of heart failure, that was published in 2021[19]. Nevertheless, as recommended in the universal definition, we only classified patients with signs or symptoms suggestive of heart failure in addition to elevated NT-proBNP levels or reduced LVEF as having coHF. For the assessment of SD, we relied exclusively on LVEF in accordance with current recommendations and did not incorporate global strain measurements, given the variability in diagnostic thresholds and limited applicability of diagnostic criteria[29]. Moreover, the proportion of patients with severe SD (LVEF < 30%) or severe DD (grade 3), and coHF was low in the investigated population[4]. Thus, the generalizability of our findings to patients with more severe cardiac dysfunction may be limited. Event numbers were modest, especially for cause‑specific readmissions and mortality, resulting in wide confidence intervals; thus, the point estimates should be interpreted cautiously and very specific outcomes as heart failure-related readmissions could not be analysed separately. Data on kidney dysfunction was not available in the SICFAIL study, although kidney dysfunction might contribute to worsening the clinical course of coHF[30]. Data on high-sensitivity Troponin was missing in nearly 20% of patients. Therefore, Troponin was analysed in a sensitivity analysis but not included in the main model. Finally, the design of the SICFAIL study does not permit conclusions regarding patients’ pre-stroke cardiac status or their long-term post-stroke cardiac trajectory, as serial echocardiographic assessments were not performed and no pre-stroke evaluation of cardiac function, based on the criteria applied in the study, was available. Accordingly, we can only hypothesise that IS contributed to cardiac dysfunction in the investigated population. We were also unable to assess temporal changes in SD and therefore cannot determine whether progression to symptomatic heart failure contributed to later events.

## 6 Conclusion

In summary, this long‑term analysis of the SICFAIL cohort demonstrated that SD and coHF identified in the acute phase of IS are powerful predictors of subsequent mortality and hospital readmission, whereas isolated DD is not. These findings support the concept of stroke–heart syndrome as a chronic phenomenon and highlight the need for integrated stroke–cardiac care. Early echocardiographic evaluation should be considered standard practice in stroke units, and patients with SD or coHF warrant adequate medical management and close follow‑up. Future multicentre studies with larger and more diverse populations are needed to validate these observations, explore the mechanisms underpinning sex and aetiology differences, and test whether targeted heart failure therapies after stroke can mitigate the substantial long‑term risks uncovered here.

## Funding sources

The SICFAIL study was funded by the German Ministry of Research and Education within the Comprehensive Heart Failure Centre Würzburg (grant numbers BMBF 01EO1004 and 01EO1504).

## CRediT authorship contribution statement

**Kathrin Ungethüm:** Writing – original draft, Visualization, Project administration, Methodology, Investigation, Formal analysis, Data curation, Conceptualization. **Felipe A. Montellano:** Writing – review & editing, Project administration, Methodology, Investigation, Data curation. **Viktoria Rücker:** Writing – review & editing, Validation, Formal analysis, Data curation. **Timo Ludwig:** Writing – review & editing, Software, Investigation, Data curation. **Charles D.A. Wolfe:** Writing – review & editing, Supervision, Methodology. **Caroline Morbach:** Writing – review & editing, Methodology, Investigation. **Stefan Frantz:** Writing – review & editing, Methodology, Investigation, Conceptualization. **Stefan Störk:** Writing – original draft, Supervision, Methodology, Investigation, Conceptualization. **Christoph Kleinschnitz:** Writing – review & editing, Supervision, Resources, Methodology, Funding acquisition, Conceptualization. **Karl Georg Haeusler:** Writing – review & editing, Supervision, Resources, Methodology, Investigation. **Peter U. Heuschmann:** Writing – original draft, Supervision, Resources, Project administration, Methodology, Investigation, Funding acquisition, Data curation, Conceptualization.

## Declaration of competing interest

The authors declare the following financial interests/personal relationships which may be considered as potential competing interests: KU is a fellow of the Graduate School of Life Sciences, University of Würzburg. FAM was supported by the German Research Foundation (Deutsche Forschungsgemeinschaft, DFG) within the UNION-CVD Clinician-Scientist Programme (project number 413657723). VR reports grants or contracts from the University Hospital Würzburg and University Hospital Essen. CDAW is funded by the National Institute for Health and Care Research (NIHR) under its Programme Grants for Applied Research (NIHR202339) and is supported by the NIHR = Applied Research Collaboration (ARC) South London at King’s College Hospital NHS Foundation Trust. The views expressed are those of the authors and not necessarily those of the NIHR = or the Department of Health and Social Care. CM reports research cooperation with the University of Würzburg and Tomtec Imaging Systems funded by a research grant from the Bavarian Ministry of Economic Affairs, Regional Development and Energy, German Germany (MED-1811–0011, LSM-2104–0002, and LSM-2403–0005); she is supported by the German Research Foundation (DFG) within the Comprehensive Research Center 1525 'Cardio-immune interfaces' (453989101, project C5) and receives financial support from the Interdisciplinary Center for Clinical Research − IZKF Würzburg (advanced clinician-scientist program; AdvCSP 3). She received advisory/speakers’ honoraria and travel grants from Tomtec, Edwards, Alnylam, Pfizer, Boehringer Ingelheim, Eli Lilly, SOBI, AstraZeneca, NovoNordisk, Alexion, Janssen, Bayer, Intellia, and EBR Systems; she is principal investigator in trials sponsored by Alnylam, Bayer, NovoNordisk, Intellia and AstraZeneca. KGH reports speakeŕs honoraria, consulting fees, lecture honoraria and/or study grants from Abbott, Amarin; Alexion, AstraZeneca, Bayer Healthcare, Biotronik, Boehringer Ingelheim, Boston Scientific, Bristol-Myers Squibb, Daiichi Sankyo, Edwards Lifesciences, Medtronic, Novartis, Pfizer, Portola, Premier Research, Sanofi, SUN Pharma, and W.L. Gore and Associates. PUH reports research grants from the European Union, German Ministry of Research and Education, German Research Foundation, Federal Joint Committee (G-BA) within the Innovation fund, German Cancer Aid, German Heart Foundation, Bavarian State, Robert Koch Institute, and University Hospital Heidelberg (within RASUNOA-prime); support from an unrestricted research grant to the University Hospital Heidelberg from Bayer, BMS, Boehringer-Ingelheim, and Daiichi Sankyo; and participation on a data and safety monitoring board in publicly funded studies (by German Research Foundation, German Ministry of Research, and Foundations). TL, SF, SS, and CK have nothing to disclose.
