# Supplementary material for: Cardiac dysfunction after acute ischaemic stroke: Long-term outcomes from the SICFAIL cohort
Source: Int J Cardiol Heart Vasc. 2026 Apr 20;64:101926. doi: 10.1016/j.ijcha.2026.101926 (PMC13122669; doi:10.1016/j.ijcha.2026.101926)
Supplement: Supplementary Data 1 [file mmc1.docx]

**SICFAIL Study Group**

Silke Wiedmann, Daniel Mackenrodt, Peter Kraft

**eMethods:** **Data collection, variable definition, and statistical analysis**

Patients

Patients with AIS according to the World Health Organization definition[1], aged 18 years or older were eligible. Study patients were examined at baseline and followed up yearly for up to five years. Patients participating in an intervention trial at the time of the index stroke were not eligible to study inclusion. The present analysis included baseline data as well as data collected during the first and second yearly follow-ups.

Procedures, Exposures and Variable Definition

During the index hospitalisation, patients underwent a standardized cardiac examination as part of clinical routine, including transthoracic and/or transoesophageal echocardiography by internally certified expert sonographers[2], 12-lead electrocardiography, and blood sampling for determination of glucose, N-terminal pro Brain natriuretic peptide (NT-proBNP), high-sensitivity Troponin T (hs-TnT), low-density lipoprotein (LDL) and HbA1c. Information on demographic background, self-reported medical history, lifestyle, HF-related symptoms, and pre-stroke needs for assistance in every-day life activities (pre-stroke dependency) were collected during a standardized interview. Additional medical data including characteristics of the index stroke, comorbidities, medication, routine laboratory analyses (glucose, lipids, HbA1c), and signs suggestive of HF were documented from structured review of medical records. Assessment of stroke aetiology (Trial of ORG 10172 in Acute Stroke Treatment (TOAST)[3]) was independently performed by three physicians[4]. Blood samples for high-sensitivity troponin T (hs-TnT) and N-terminal pro B-type natriuretic peptide (NT-proBNP) were drawn at baseline, stored at -80°C and analysed at one single time-point after completion of recruitment[4].

The investigation of SD and DD was based on echocardiographic examination according to current guidelines[5,6]. Patients with SD were excluded from analyses regarding DD. As more than 95% of DD patients within the SICFAIL cohort had Grade 2 or Grade 3 DD[4], no distinction of DD grades was made due to power issues. For the definition of HF, signs and symptoms from self-report and clinical diagnostics of HF and NT-proBNP levels in combination with echocardiographic findings were respected[7]. The detailed diagnostic algorithms for SD, DD and HF used in the SICFAIL study[4] are given in Table 1*.* Hypertension was defined as self-reported hypertension or intake of antihypertensive medication. Diabetes mellitus was defined by self-report, elevated HbA1c ≥6.5% (48 mmol/mol) at admission, or intake of antidiabetic medication. Alterations in lipid metabolism were defined as a self-reported history in hypercholesterinaemia, intake of lipid-lowering medication or LDL levels ≥130 mg/dl at admission.

Follow-up and outcome

Information on readmission (due to recurrent stroke, myocardial infarction, arrhythmia, heart failure, intracerebral haemorrhage, atrial fibrillation, or other diagnosis) was collected yearly by phone or mail using a standardized questionnaire. If a patient could not be reached, information on vital status was collected by a patient’s next of kin or the local resident’s registration office. The main outcome investigated in this study was the combined endpoint of all-cause mortality and all-cause readmission. Secondary analyses were performed to investigate single endpoints (all-cause mortality, all-cause readmission, CVD-related readmission (stroke or cardiac event), heart-related readmission (cardiac event), stroke-related readmission). During follow-up interviews, upon learning abouts a patient’s decease, we refrained from further questions including cause of death or readmission. For this reason, readmission was not considered in the analyses in patients who died within the two-year follow-up period.

Analysed patient population and censoring

Patients who withdraw consent or could not be reached during follow-up were excluded from analyses of the primary endpoint. Patients who withdraw their consent during the second-year follow-up were censored from the analyses after 24 months, patients who could not be re-contacted, or had missing information on readmission during the second-year follow-up, were censored from the analyses after 12 months. Patients without valid echocardiography data or insufficient outcome information were excluded. To analyse all-cause mortality as a secondary outcome, all patients with information on vital status were included.

In case of a patient’s outcome event without information on the date of event, the time of event was set to the last attempt of contact after 12 months (first-year follow-up), or after 24 months (second-year follow-up), respectively. If only the year, but no exact date of death was known, time of death was set to 31^st^ December.

Statistical Analyses

In line with the Strengthening the Reporting of Observational Studies in Epidemiology (STROBE) recommendations, no statistical tests to analyse differences of baseline characteristics between patient groups are reported[8].

Several sensitivity analyses comprised:

1. *Inclusion of Biomarker*

The impact of biomarkers of high-sensitivity Troponin above the prespecified cut-off of 14 ng/ml was investigated by separate inclusion in the completely adjusted Model 2.

1. *Effect modification by sex, coronary heart disease and cardiovascular risk factors*

Reacting to increasing evidence of sex-specific differences in the heart-brain interaction[9], a potential effect modification by sex using interaction terms of sex and systolic dysfunction was tested in Model 1. Further potential effect modifications were tested using interaction terms of coronary heart disease, hypertension, dyslipidaemia, and diabetes with systolic dysfunction.

1. *Exclusion of patients without exact information on the time of event*

To analyse a potential statistical distortion due to the imputation of time to event (12 or 24 months) in patients where no exact date of the outcome occurrence was available, a sensitivity analysis was performed in Model 2, excluding all patients with imputed time to outcome occurrence.

**Supplemental Table 1. Distribution of the combined endpoint within two years after the index event.**

|  | All  (n=554) | Patients with SD  (n=45) | Patients with DD  (n=109) | Patients with HF  (n=33) |
| --- | --- | --- | --- | --- |
| Readmission, n (%) | 206 (37.2%) | 24 (66.7) | 39 (40.2) | 18 (62.1) |
| CVD | 81 | 12 | 17 | 6 |
| Cardiac Event | 34 | 6 | 5 | 4 |
| Myocardial Infarction | 9 | 1 | 2 | 1 |
| Heart Failure | 7 | 1 | 1 | 1 |
| Angina Pectoris | 9 | 0 | 2 | 1 |
| Heart rhythm abnormalities | 15 | 4 | 2 | 1 |
| Stroke | 56 | 7 | 14 | 4 |
| Other reason | 135 | 12 | 22 | 14 |
| All-cause mortality, n (%) | 63 (11.4%) | 9 (20.0) | 19 (17.4) | 6 (18.2) |
| Readmission or all-cause Mortality, n (%) | 269 (48.6%) | 33 (73.3) | 53 (48.6) | 24 (72.7) |

**Supplemental Table 2. Non-responder Analysis**

|  | **Responder**  **(n=554)** | **Non-responder  (n=90)** | **p-value** |
| --- | --- | --- | --- |
| Age, median (IQR) | 70.5  (60.0-78.0) | 73.5 (61.0-78.0) | 0.33 |
| <55 years, n (%) | 88 (15.9) | 14 (15.6) | 0.50 |
| 55-64 years, n (%) | 100 (18.1) | 15 (16.7) |  |
| 65—74 years, n (%) | 145 (26.2) | 17 (18.9) |  |
| 75-84 years, n (%) | 166 (30.0) | 34 (37.8) |  |
| ≥85 years, n (%) | 55 ((9.9) | 10 (11.1) |  |
| Male, n (%) | 343 (61.9) | 53 (58.9) | 0.58 |
| Dependency, pre-stroke, n (%) | 65 (12.0) | 15 (17.7) | 0.14 |
| NIHSS, median (IQR) | 3 (1-5) | 2 (1-4.5) | 0.06 |
| ≤4 | 402 (72.6) | 66 (75.0) | 0.58 |
| 5-15 | 135 (24.4) | 21 (23.9) |  |
| ≥16 | 17 (3.1) | 1 (1.1) |  |
| Thrombolysis, n (%) | 109 (19.7) | 15 (16.7) | 0.50 |
| Mechanical Thrombectomy, n (%) | 7 (1.3) | 1 (1.1) | >0.99 |
| TOAST classification, n (%) |  |  | 0.34 |
| Large-artery artherosclerosis, | 64 (11.6) | 8 (8.9) |  |
| Cardioembolic stroke | 161 (29.1) | 31 (34.4) |  |
| Small vessel occlusion | 81 (14.6) | 12 (13.3) |  |
| Stroke of other determined cause | 44 (7.9) | 7 (7.8) |  |
| Undetermined aetiology | 203 (36.6) | 32 (35.6) |  |
| Time from onset to admission >6 hours | 259 (46.8) | 53 (58.9) | **0.03** |
| ***Comorbidities / Medical History*** | | |  |
| Atrial fibrillation, n (%) | 119 (21.5) | 24 (16.7) | 0.27 |
| Hypertension, n (%) | 374 (67.5) | 59 (65.6) | 0.71 |
| History in Hypercholesterinaemia or LDL>130 mg/dl at baseline, n (%) | 360 (68.8) | 51 (63.8) | 0.36 |
| Diabetes, n (%) | 152 (28.3) | 35 (40.7) | **0.02** |
| History in stroke / TIA, n (%) | 128 (23.4) | 25 (30.1) | 0.19 |
| Coronary heart disease, n (%) | 91 (16.7) | 11 (13.1) | 0.40 |
| ***Cardiac dysfunction*** | | |  |
| Systolic dysfunction, n (%) | 45 (8.1) | 17 (18.9) | **<0.01** |
| Diastolic dysfunction*, n (%) | 109 (22.7) | 19 (27.5) | 0.38 |
| Clinically overt heart failure, n (%) | 33 (6.0) | 2 (2.2) | 0.21 |

**Supplemental Table 3.** **Univariate analysis of the association of different types of cardiac dysfunction with primary and secondary endpoints. The HR and 95% Confidence Intervals presented were calculated using Cox proportional hazard logistic regression models.**

|  | **Univariate** |
| --- | --- |
| ***Primary Endpoint*** | |
| **Combined Endpoint** |  |
| Systolic Dysfunction | **2.13 (1.48-3.07)** |
| Diastolic Dysfunction | 1.19 (0.87-1.62) |
| Clinically Overt Heart Failure | **2.17 (1.42-3.30)** |
| ***Secondary Endpoints*** | |
| **All-cause Mortality** |  |
| Systolic Dysfunction | 1.95 (0.96-3.95) |
| Diastolic Dysfunction | **2.20 (1.24-3.91)** |
| Clinically Overt Heart Failure | 1.82 (0.79-4.22) |
| **All-cause Readmission** | |
| Systolic Dysfunction | **1.89 (1.24-2.90)** |
| Diastolic Dysfunction | 1.01 (0.71-1.44) |
| Clinically Overt Heart Failure | **1.87 (1.15-3.03)** |
| **Readmission for CVD** | |
| Systolic Dysfunction | **2.29 (1.24-4.24)** |
| Diastolic Dysfunction | 1.35 (0.77-2.35) |
| Clinically Overt Heart Failure | 1.32 (0.58-3.04) |
| **Readmission for a Cardiac Event** | |
| Systolic Dysfunction | 2.12 (0.74-6.08) |
| Diastolic Dysfunction | 0.94 (0.35-2.51) |
| Clinically Overt Heart Failure | 2.04 (0.62-6.73) |
| **Stroke-related Readmission** | |
| Systolic Dysfunction | 1.78 (0.80-3.92) |
| Diastolic Dysfunction | 1.67 (0.87-3.13) |
| Clinically Overt Heart Failure | 1.22 (0.44-3.37) |

**Supplemental Table 4. Raw model cefficients without consideration of cardiac function. Multivariable Analysis of confounders with the combined primary endpoint of all-cause readmission and all-cause mortality. The Hazard Ratio (HR) and 95% Confidence Intervals (CI) presented were calculated in Cox proportional hazard logistic regression models.**

| ***Model 1*** | *HR 95% CI* |
| --- | --- |
| Age, per year | **1.01 (1.00-1.02)** |
| NIHSS, per point | **1.03 (1.00-1.06)** |
| Dependency pre-stroke (yes vs. no) | 1.42 (0.99-2.01) |
| ***Model 2*** |  |
| Age, per year | 1.00 (0.99-1.01) |
| NIHSS, per point | 1.03 (0.99-1.06) |
| Dependency pre-stroke (yes vs. no) | 1.24 (0.85-1.81) |
| History in stroke/TIA (yes vs. no) | 1.41 (1.06-1.87) |
| Atrial fibrillation (yes vs. no) | 1.30 (0.86-1.97) |
| History in coronary heart disease (yes vs. no) | 1.45 (1.06-1.99) |
| Diabetes mellitus (yes vs. no) | 1.19 (0.90-1.57) |
| Hypertension (yes vs. no) | 1.25 (0.87-1.80) |
| Dyslipidaemia (yes vs. no) | 1.09 (0.80-1.47) |
| Stroke aetiology (cardioembolic vs. non-cardioembolic) | 1.02 (0.70-1.47) |


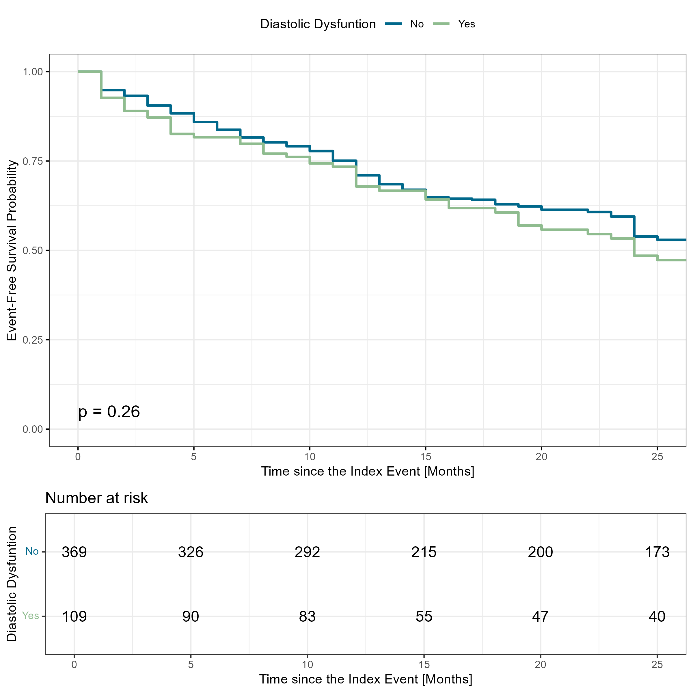

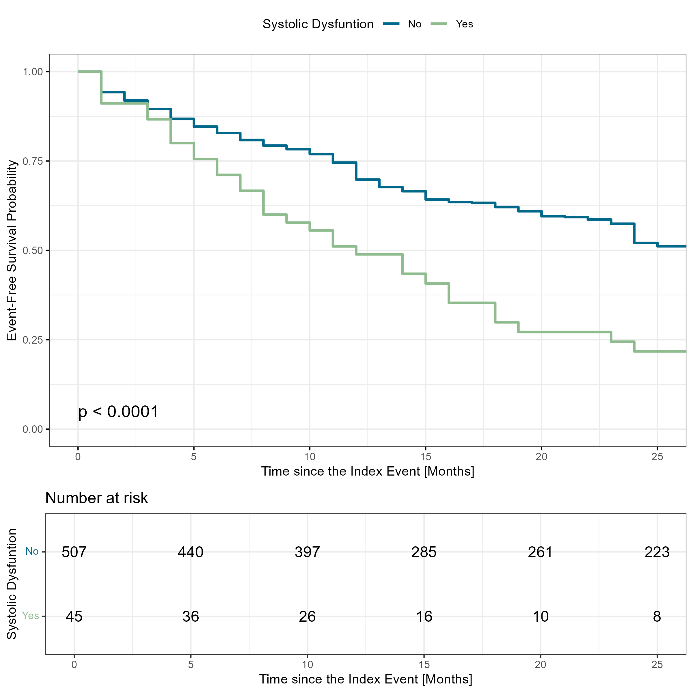


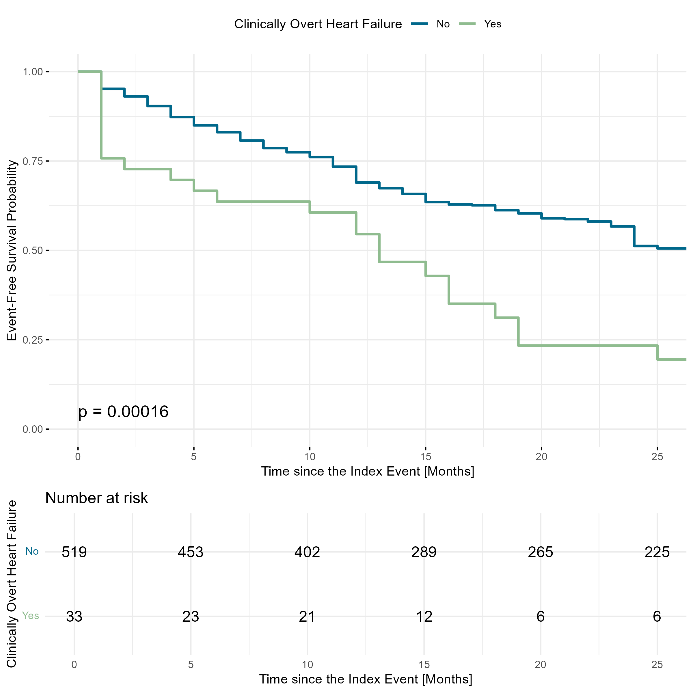


**Supplemental Figure 1. Kaplan-Meier-Curves for the association of the composite of all-cause readmission and all-cause mortality with different types of cardiac dysfunction.**

**References**

[1] S. Hatano, Experience from a multicentre stroke register: a preliminary report, Bull World Health Organ 54 (1976).

[2] C. Morbach, G. Gelbrich, M. Breunig, T. Tiffe, M. Wagner, P.U. Heuschmann, S. Störk, Impact of acquisition and interpretation on total inter-observer variability in echocardiography: results from the quality assurance program of the STAAB cohort study, Int. J. Cardiovasc. Imaging 34 (2018) 1057–1065. https://doi.org/10.1007/s10554-018-1315-3.

[3] H.P. Adams, B.H. Bendixen, L.J. Kappelle, J. Biller, B.B. Love, D.L. Gordon, E.E. Marsh, Classification of subtype of acute ischemic stroke. Definitions for use in a multicenter clinical trial. TOAST. Trial of Org 10172 in Acute Stroke Treatment., Stroke 24 (1993) 35–41. https://doi.org/10.1161/01.STR.24.1.35.

[4] P.U. Heuschmann, F.A. Montellano, K. Ungethüm, V. Rücker, S. Wiedmann, D. Mackenrodt, A. Quilitzsch, T. Ludwig, P. Kraft, J. Albert, C. Morbach, S. Frantz, S. Störk, K.G. Haeusler, C. Kleinschnitz, Prevalence and determinants of systolic and diastolic cardiac dysfunction and heart failure in acute ischemic stroke patients: The SICFAIL study, ESC Heart Fail. 8 (2021) 1117–1129. https://doi.org/10.1002/ehf2.13145.

[5] R.M. Lang, L.P. Badano, V. Mor-Avi, J. Afilalo, A. Armstrong, L. Ernande, F.A. Flachskampf, E. Foster, S.A. Goldstein, T. Kuznetsova, P. Lancellotti, D. Muraru, M.H. Picard, E.R. Rietzschel, L. Rudski, K.T. Spencer, W. Tsang, J.-U. Voigt, Recommendations for Cardiac Chamber Quantification by Echocardiography in Adults: An Update from the American Society of Echocardiography and the European Association of Cardiovascular Imaging, Eur. Heart J. – Cardiovasc. Imaging 16 (2015) 233–271. https://doi.org/10.1093/ehjci/jev014.

[6] S.F. Nagueh, O.A. Smiseth, C.P. Appleton, B.F. Byrd, H. Dokainish, T. Edvardsen, F.A. Flachskampf, T.C. Gillebert, A.L. Klein, P. Lancellotti, P. Marino, J.K. Oh, B. Alexandru Popescu, A.D. Waggoner, Houston, Texas; Oslo, Norway; Phoenix, Arizona; Nashville, Tennessee; Hamilton, Ontario, Canada; Uppsala, Sweden; Ghent and Liège, Belgium; Cleveland, Ohio; Novara, Italy; Rochester, Minnesota; Bucharest, Romania; and St. Louis, Missouri, Recommendations for the Evaluation of Left Ventricular Diastolic Function by Echocardiography: An Update from the American Society of Echocardiography and the European Association of Cardiovascular Imaging, Eur. Heart J. – Cardiovasc. Imaging 17 (2016) 1321–1360. https://doi.org/10.1093/ehjci/jew082.

[7] P. Ponikowski, A.A. Voors, S.D. Anker, H. Bueno, J.G.F. Cleland, A.J.S. Coats, V. Falk, J.R. González-Juanatey, V.-P. Harjola, E.A. Jankowska, M. Jessup, C. Linde, P. Nihoyannopoulos, J.T. Parissis, B. Pieske, J.P. Riley, G.M.C. Rosano, L.M. Ruilope, F. Ruschitzka, F.H. Rutten, P. Van Der Meer, 2016 ESC Guidelines for the diagnosis and treatment of acute and chronic heart failure: The Task Force for the diagnosis and treatment of acute and chronic heart failure of the European Society of Cardiology (ESC)Developed with the special contribution of the Heart Failure Association (HFA) of the ESC, Eur. Heart J. 37 (2016) 2129–2200. https://doi.org/10.1093/eurheartj/ehw128.

[8] J.P. Vandenbroucke, E. Von Elm, D.G. Altman, P.C. Gøtzsche, C.D. Mulrow, S.J. Pocock, C. Poole, J.J. Schlesselman, M. Egger, Strengthening the Reporting of Observational Studies in Epidemiology (STROBE): Explanation and Elaboration, Epidemiology 18 (2007) 805–835. https://doi.org/10.1097/EDE.0b013e3181577511.

[9] A. Rossi, N. Mikail, S. Bengs, A. Haider, V. Treyer, R.R. Buechel, S. Wegener, K. Rauen, A. Tawakol, C.N. Bairey Merz, V. Regitz-Zagrosek, C. Gebhard, Heart–brain interactions in cardiac and brain diseases: why sex matters, Eur. Heart J. 43 (2022) 3971–3980. https://doi.org/10.1093/eurheartj/ehac061.
